# Correction: The effects of icon design features on user perception and preference: A case study of icons for Covid-19

**DOI:** 10.1371/journal.pone.0328791

**Published:** 2025-07-21

**Authors:** The PLOS One Staff

The Funding statement for this article is incorrect. The correct Funding statement is as follows: Guangdong Province Education Science 13th Five-Year Plan 2020 Research Special Program (2020GXJK262 to QL), Guangdong Province 2021 General Colleges and Universities Characteristic and Innovative Projects (2021KTSCX260 to QL). The funders had no role in study design, data collection and analysis, decision to publish, or preparation of the manuscript.

The publisher apologizes for the error.
