# Supplementary material for: The effects of icon design features on user perception and preference: A case study of icons for Covid-19
Source: PLoS One. 2024 Sep 3;19(9):e0305290. doi: 10.1371/journal.pone.0305290 (PMC11371222; doi:10.1371/journal.pone.0305290)
Supplement: Attachment — Submitted filename: Review PONE-D-23-31275.docx [file pone.0305290.s004.docx]

PONE-D-23-31275

The study entitled: “The effects of icon design features on user perception and preference: A case study of icons for Covid-19” is an interesting study with some good stimulus material and good data collection. However, there are number of problems that would prevent publication of this study in Plos One. The major problem is a hodge podge of statistics that are in search of the question. The author could have and should have been able to determine which icons were most effective on their three dimensions within the group (e.g. for example for handwashing or mask wearing). That would have been useful information. So, I will begin with the major problems with the results and then discuss other problems.

RESULTS

The results need a major overhaul. The result lack statistical tests and most of the results are subjective and obtained through “eyeballing.”

1. The authors could have done a factor analysis and computed reliability coefficients for each of the 3 variables: communication effectiveness, design quality, and semantic distance but this is missing. The authors have psychometrically valid data for each of these dimensions but they do not use it. Since each of these scales contain multiple semantic differential items that represent the dimension an internal reliability coefficient is necessary and easy to compute. From that point some meaningful statistical tests could have been conducted and reported.
2. We need to know if results for each dimension were aggregated and divided by the number of items that comprise the dimension or what. I cannot find scores for each dimension. This needs major revision and clarification.
3. Just reporting the top 15 icon function types is subjective; were these significantly different?
4. What does it tell is that some function types are better than others? Does it mean that we should tell people to wash hands but not check their temperature? The research question needs to be posed and then systematically answered.
5. We need statistical evidence to back the authors’ assertion that: “it can be initially determined that users prefer icons that contain specific character images, actions.”
6. Similarly, the authors assert that “It is suggested that users prefer icons associated with images when real or illustrated more concretely.” What statistical evidence supports this assertion?
7. What would be interesting would be to ascertain for each type of icon which was significantly better than others for that types. Which hand washing icon for example was the most effective using statistical tests for each dimension.
8. The authors collected some good data using rigorous data collection techniques but there is no rigorous analysis of the data as a reader would expect.
9. For aesthetics the authors state: “In terms of mean values, all samples scored between 5 and 6 (out of a total of 7) for the subjective design features, the distribution of scores over the mean did not differ much…” But without statistical tests, how do we know this is true?
10. In the correlation analysis it is unclear as to what is being correlated. This needs some major revision.
11. Ranks need some special statical procedures to deal with ordinal data.

ICONS

The authors did an excellent job of 120 Icons for the study that represent a number of important Covid prevention behaviors. Similarly, it is impressive that Icons were obtained from 26 countries potentially providing some cross cultural validity.

THEORETICAL BASE

The article need a broader theoretical base from the study of human communication to understand the basis of iconic communication. See a number of books on nonverbal communication for the conceptual and neurophysiological basis for iconic communication. The authors should provide at least a brief summary of literature on the value of icons from the literature of nonverbal communication. Their history and conceptual basis goes way beyond computer screens-they were used and studies in many contexts (e.g. traffic signs, medicine bottles, maps, product branding etc.) long before the advent of computer screens.

SAMPLE

Questionnaires were distributed sing the Star App buy we do not know if the participants were Chinese, European, American, global or what. This need clarification.

The authors provide a good table on characteristics of participants and good age distribution. However, we still do not know where they are from? Were these all from on particular country or was it an international sample? Was it a general sample of the Questionnaire Star App, or were some parameters specified. More detail is required.
