# Supplementary material for: The effects of icon design features on user perception and preference: A case study of icons for Covid-19
Source: PLoS One. 2024 Sep 3;19(9):e0305290. doi: 10.1371/journal.pone.0305290 (PMC11371222; doi:10.1371/journal.pone.0305290)
Supplement: Attachment — Submitted filename: Reviewer 02- Response to the Comments.docx [file pone.0305290.s005.docx]

**Response to the Comments of the Reviewers**

Manuscript ID: PONE-D-23-31275

Dear Reviewers:

Thank you for your comments concerning our manuscript entitled “**The effects of icon design features on user perception and preference: A case study of icons for Covid-19**”. Those comments are all valuable and very helpful for revising and improving our paper. We have studied comments carefully and have made correction which we hope to meet with approval. The corrections and our reply to reviewer’s comments are marked in **blue color** in the revised manuscript for convenience. The main corrections in the paper and the responds to the reviewer’s comments are as flowing:

**Reviewer #2:**

Congratulations to the authors for their work on this study. The paper offers valuable insights into icon evaluation in public health. However, I have several comments and recommendations:

**Introduction:**

- Deepen the exploration of how iconography impacts public health communication. Highlight the influence of icon design on public behavior and decision-making, particularly during health crises.

**Reply:** Thank you very much for pointing out the problem. We are taking your suggestions further to explore how iconography can influence public health communication, especially during health crises. Please check **p2-p3** for more details.

- Incorporate a concise review of previous studies on public health iconography, emphasizing the novel contributions or challenges your research presents.

**Reply:** Thank you for your constructive comments, we have added research literature related to public health iconography in the second half of the introduction and emphasized the contributions and challenges of this study, please check **p3-p5** for more details.

- Clearly articulate the theoretical framework guiding your study, linking it directly to your objectives and anticipated results.

**Reply:** Thanks to your comments, we have added relevant research literature to the first subsection of the literature review (iconic communication) and briefly stated the theoretical basis of iconic communication from the perspective of semiotics and cognitive psychology, as detailed on **page 5- 8**.

Section 2.3 Evaluation Metrics for Icon Design:

- The categorization in Table 3 is somewhat unclear, particularly regarding the second and third categories. I recommend revising the descriptions to ensure they align with the text and avoid repetition.

**Reply:** Thank you for your suggestion. The description of "design quality" is more general and seems to encompass all three, so we have changed the original "Design Quality" to **"Visual Design Perception"** for clarity. Please check **page19** and **Table 3** for more details.

**Methodology:**

- Clarify the criteria for selecting icons and the reasoning behind these choices to enhance the study's replicability.

**Reply:** Thank you very much for pointing out the issue.In order to be more representative and replicable, icons representing different design types, Image-related, Concept-related, Arbitrary, Semi-abstract, and Combined, were purposely selected for this study, excluding single textual and abbreviated types. In addition, textual descriptions were removed from the Combined icons when designing the questionnaire. Please check **page15** for more details.

- Provide a more detailed account of participant demographics to strengthen the study's validity.

**Reply:** Thanks for your suggestion, we've added a detailed description of our demographic data as below:

“Table 1 shows the demographic characteristics of the participants. A total of 300 participants were recruited to participate in the study, all of whom were residents of different parts of mainland China. There was a balanced proportion of men and women among the participants, with men (n=102) accounting for 45.7% and women (n=121) accounting for 54.3%; the participants were all over 18 years old, and most of the participants were concentrated in the younger age group of 18-45 years old (n=152), which accounted for 68.2% of the participants; the participants' education level was concentrated in college, undergraduate and graduate students (n=185), which accounted for 82.9%, the good educational background indicates that the participant group has a good cognitive ability of icon design.”

Please see manuscript **P14-15** and **Table 1** for details.

- Detail the validation or pilot phase of the Questionnaire Star App, including languages used, and add relevant references.

**Reply:** Thank you for your constructive comments. It is very helpful to us. We have corrected the issues you raised in this subsection on **Participants**. Please see **page 13**.

- Explain the rationale for choosing the Bentler and Chou method.

**Reply:** Thank you for your comments. The rationale for the selection of the Bentler and Chou methods was explained as follows:

“Since the population of the parent group could not be determined, Bentler and Chou's method of determining sample size was used in this study. According to Bentler and Chou , the sample size is 5-10 times the number of questionnaire items when the parent group of the population is unknown, and the sample size can be enlarged by 20% by considering the number of invalid samples[42].”

Please see manuscript **P13-14** for details.

- Clarify the process and purpose of the ranking test. Table 2 appears to present results rather than methodology, which could be confusing.

**Reply:** Yes, in order not to duplicate the presentation of all selected icons, we use a table (Table 2) to exhibit the 120 icons selected for the study and the results of the sorting test.

- Elaborate on the use of factor analysis and PCA, as they were mentioned but not detailed in the paper.

**Reply:**Thank you very much for pointing out the problem, we have explained in detail in subsection **Statistical analysis** the reasons for using factor analysis and principal component analysis in this study. Please check the subsection **Statistical analysis**, **page 21-22.**

- For Table 2's categorization, consider supporting your approach with robust strategies like cluster analysis, Multidimensional Scaling (MDS), Content Analysis, or advanced AI techniques like CNNs, Autoencoders, Transfer Learning, or Deep Learning with Data Augmentation.

**Reply:** Thank you for your suggestion. However, given the time and length constraints, as well as in terms of the objectives and questions of this study, we focused on the second testing phase (subjective scoring test).

- Overall, a more thorough statistical analysis plan is needed, outlining the methods for data analysis and their alignment with your research questions.

**Reply:** Thank you for pointing out the shortcomings, we have added as much data analysis as possible to support our research goals and questions. Please see manuscript **P23-30** for more details.

We hope that the revised manuscript will better reflect the value of the study.
